# Alpha-Synuclein Autoimmune Decline in Prodromal Multiple System Atrophy and Parkinson’s Disease

**DOI:** 10.3390/ijms23126554

**Published:** 2022-06-12

**Authors:** Jonas Folke, Emil Bergholt, Bente Pakkenberg, Susana Aznar, Tomasz Brudek

**Affiliations:** 1 Centre for Neuroscience & Stereology, Department of Neurology, Copenhagen University Hospital, Bispebjerg-Frederiksberg Hospital, DK-2400 Copenhagen NV, Denmark; emil_bergholt@hotmail.com (E.B.); bente.pakkenberg@regionh.dk (B.P.); susana.aznar.kleijn@regionh.dk (S.A.); tomasz.brudek@regionh.dk (T.B.); 2 Copenhagen Center for Translational Research, Copenhagen University Hospital, Bispebjerg-Frederiksberg Hospital, DK-2400 Copenhagen NV, Denmark; 3 Institute of Clinical Medicine, Faculty of Health, University of Copenhagen, DK-2100 Copenhagen Ø, Denmark

**Keywords:** multiple system atrophy, Parkinson’s disease, naturally occurring autoantibodies (nAbs), alpha-synuclein, prodromal

## Abstract

Multiple-system trophy (MSA) and Parkinson’s Disease (PD) are both progressive, neurodegenerative diseases characterized by neuropathological deposition of aggregated alpha-synuclein (αSyn). The causes behind this aggregation are still unknown. We have reported aberrancies in MSA and PD patients in naturally occurring autoantibodies (nAbs) against αSyn (anti-αSyn-nAbs), which are important partakers in anti-aggregatory processes, immune-mediated clearance, and anti-inflammatory functions. To elaborate further on the timeline of autoimmune aberrancies towards αSyn, we investigated here the Immunoglobulin (Ig) affinity profile and subclass composition (IgG-total, IgG1-4 and IgM) of anti-αSyn-nAbs in serum samples from prodromal (p) phases of MSA and PD. Using an electrochemiluminescence competition immunoassay, we confirmed that the repertoire of high-affinity anti-αSyn-nAbs is significantly reduced in pMSA and pPD. Further, we demonstrated that pPD had increased anti-αSyn IgG-total levels compared to pMSA and controls, concordant with increased anti-αSyn IgG1 levels in pPD. Anti-αSyn IgG2 and IgG4 levels were reduced in pMSA and pPD compared with controls, whereas anti-αSyn IgG3 levels were reduced in pMSA compared to pPD and controls. The results indicate that the impaired reactivity towards αSyn occurs prior to disease onset. The apparent lack of high-affinity anti-αSyn nAbs may result in reduced clearance of αSyn, leading to aggregation of the protein. Thus, this study provides novel insights into possible causes behind the pathogenesis in synucleinopathies such as MSA and PD.

## 1. Introduction

The multiple-system atrophy (MSA) and Parkinson’s disease (PD) are both progressive, neurodegenerative disorders characterized by abnormal misfolding and depositions of alpha-synuclein (αSyn) into neurons in PD, and into oligodendrocytes in MSA [1,2]. For this reason, they are defined as synucleinopathies, a group of diseases characterized by pathological αSyn aggregates in brain and peripheral neuronal cells, which also include dementia with Lewy bodies (DLB) and the rarer pure autonomic failure (PAF) [3]. There is accumulating evidence indicating that αSyn deposition not only occurs in the brain but to a high degree also in peripheral organs. This is shown by increased phosphorylation of αSyn at Serine-129 αSyn in skin samples [4,5], αSyn accumulation in the gut [6,7,8], as well as a generalized whole-body αSyn-seeding capability in MSA and PD [9,10,11]. Remarkably, αSyn can also be detected in different body fluids such as blood, cerebrospinal fluid, saliva, and as shown most recently in tears [12,13,14], which are body compartments accessible to the immune system. Synucleinopathies can thereby be considered multisystem disorders, substantiating the hypothesis that the αSyn pathology spreading from peripheral organs to the brain may be a critical feature at the prodromal phases before the classical, motor systems get manifested.

Naturally occurring autoantibodies (nAbs), mainly immunoglobulin (Ig) G and IgM isotypes, comprise an important part of the Ig repertoire from the innate immune system. nAbs have protective roles in maintaining protein homeostasis via a T-cell-independent manner [15,16]. As shown in pre-clinical models, anti-αSyn nAbs isolated from intravenous Ig (IVIg) confer protective properties [17], presenting highly diverse binding profiles to monomeric, oligomeric, and fibrillary structures of αSyn [17]. Furthermore, specific nAbs produced from single αSyn clonotypes from healthy individuals also display protective effects [18]. In humans, we and others have evaluated the IgG repertoire of αSyn reactive nAbs in PD and MSA [19,20,21,22], showing specifically in PD a tendency to increased total IgG anti-αSyn nAbs levels, particular in early disease as also reviewed by Scott et al. 2018 [22]. The complexity in αSyn autoimmunity further expands, since the total IgG anti-αSyn nAbs compartment consists of four subclasses, IgG1–4, each of which have different biological functions [23]. We have previously found several aberrancies in the four IgG subclasses and IgM, both in plasma and CSF, which is suggestive of highly and perhaps changing immuno-αSyn interactions and responses. Most importantly, when looking at the nAbs affinity, we found a decline in the high-affinity repertoire of anti-αSyn nAbs in PD and MSA patients, as shown both for plasma [24] and CSF [21]. The progressive nature of synucleinopathies and previous reports of phenoconversion from prodromal syndromes such as idiopathic REM-sleep behavior disorder (iRBD) [25] to synucleinopathic disorders serve as evidence that pathological events occur years prior to symptoms onset. It is our belief that this αSyn-specific immune decline may have a pathological effect on αSyn homeostasis and impair the clearance of αSyn toxic species and for that reason, we hypothesize that the reduction of high-affinity anti-αSyn-nAbs occurs years prior to disease onset. The aim of this study was therefore to evaluate high-affinity anti-αSyn nAbs in serum samples taken from PD and MSA patients up to 12 years before diagnosis. A reduction of high-affinity anti-αSyn-nAbs in the prodromal phases advocates for immunization strategies already administrated according to prodromal symptoms/phases.

## 2. Results

### 2.1. Affinity Measurement of Anti-αSyn nAbs in pMSA and pPD

**Figure 1:**
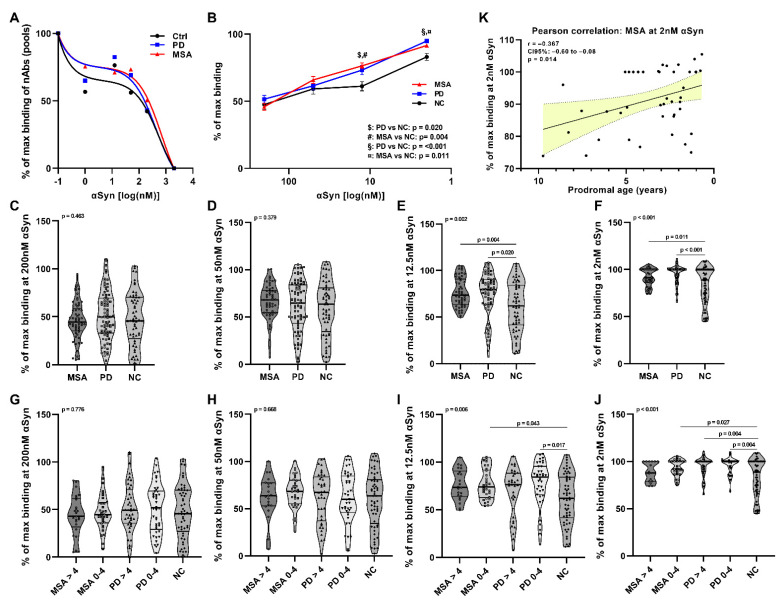
Serum anti-αSyn nAb affinity profiles. (**A**) A two-site inhibition curve profiles of pooled plasma samples from 10 age- and sex-matched serum samples from prodromal (p)MSA (red triangles and line), pPD (blue squares and line), and controls (black circles and line). Binding profiles of anti-αSyn nAbs in individual pMSA (*n* = 59), pPD (*n* = 82) and control (*n* = 67) serum samples are summarized in (**B**). Binding of serum anti-αSyn Nabs to immobilized αSyn monomers in competitive immunoassay in the presence of (**C**) 200 nM, (**D**) 50 nM, (**E**) 12.5 nM, and (**F**) 2 nM free αSyn. (**G**–**J**) shows the data from (C–F) for individual serum samples from pMSA and pPD divided into two prodromal age stages (0–4 years and > 4 years prior to diagnosis), Pearson’s correlation of prodromal age, and the high-affinity nAbs at 2 nM in pMSA patients (**K**). All data are presented as “% of max binding” of anti-αSyn nAbs calculated as described in Section 4.5. Differences between groups were tested using multiple linear regression modeling including age and sex as confounding variables. Graphs (C–J) are presented as truncated violin plots with median (bold lines) and 25% quantiles (thin lines).

**Figure 2:**
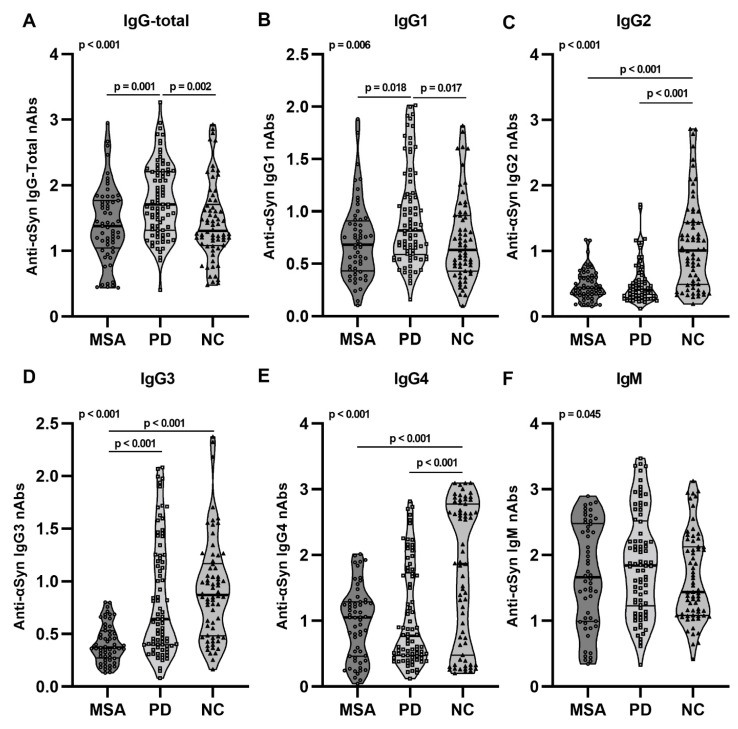
Serum anti-αSyn IgG-total, IgG subclasses, and IgM nAbs. The graphs represent relative levels of anti-αSyn (**A**); IgG-total and IgG1–4 (**B**–**E**); and IgM (**F**) nAbs in individual pMSA (*n* = 59), pPD (*n* = 82) and control (*n* = 67) serum samples. Data are presented as relative optical densities normalized to standard curve using anti-αSyn monoclonal antibody. Differences between groups were tested using multiple linear regression modeling including age and sex as confounding variables. Graphs are presented as truncated violin plots with median (bold lines) and 25% quantiles (thin lines).

To measure the relative affinity of anti-αSyn IgG nAbs in serum, we adapted our previously established competitive ELISA assay [21,24] to the ultrasensitive Meso Scale Discovery (MSD) platform. The anti-αSyn binding profiles were firstly assessed using randomly chosen serum pooled samples of 10 pMSA and 10 pPD patients, and 10 controls. We observed noticeable differences in the binding profiles between control, and pMSA and pPD patients (Figure 1A). The double sigmoid profile divides the anti-αSyn nAbs into two distinct subsets; the low-affinity anti-αSyn nAbs are revealed in the presence of high concentrations of αSyn monomers (25–200 nM), and the high-affinity nAbs are revealed in the presence of low concentrations of αSyn monomers (1–25 nM). Based on the competition curve profile, we selected four different concentrations of αSyn to measure the relative affinity of anti-αSyn nAbs (high-affinity profile: 2 nM and 12.5 nM αSyn; low-affinity profile: 50 nM and 200 nM αSyn) in the individual serum samples from 59 pMSA and 82 pPD patients, and 67 controls. The repertoires of high-affinity nAbs were significantly different at 12.5 nM (Figure 1B,E) and 2 nM (Figure 1B,F) between controls and pMSA (12.5 nM: *p* = 0.004, 2 nM: *p* = 0.011) and pPD (12.5 nM: *p* = 0.020; 2 nM: *p* < 0.001), respectively. Comparing the groups separated based on time prior to diagnosis (0–4 years and >4 years prior to diagnosis), we observed a significant reduction in anti-αSyn nAbs in pMSA at 12 nM (*p* = 0.043) and 2 nM (*p* = 0.027) 0–4 years prior to diagnosis compared to controls. In pPD we observed a reduction at 12 nM 0–4 years prior to diagnosis (*p* = 0.017) and a reduction at 2 nM in pPD at >4 years prior (*p* = 0.004) and 0–4 years prior (*p* = 0.004) compared to controls. Using linear regression analyses, only pMSA at 2 nM showed a significant decrease of high-affinity nAbs correlated with the prodromal age (Pearson’s *r* = −0.367, CI95%: −0.60 to −0.08, *p* = 0.014) (Figure 1K). All statistics and correlation analyses are summarized in Tables S1 and S2, respectively.

### 2.2. Anti-αSyn IgG Subclasses and IgM nAbs in pMSA and pPD

Given that different antibody IgG isotypes (IgG-total, IgG1-4) and IgM carry out different functions in the immune system, we further aimed to explore the autoimmune reactivity towards αSyn in the prodromal phases of MSA and PD. We observed a significant increase of total IgG reactivity towards αSyn in pPD compared to pMSA (*p* = 0.001) and controls (*p* = 0.002) (Figure 2A). This increase was traced down to increased anti-αSyn IgG1 nAbs in pPD compared to both pMSA (*p* = 0.018) and controls (*p* = 0.017) (Figure 2B). Interestingly, we observed similar patterns with reduced nAb levels in pMSA and pPD compared to controls in the anti-αSyn IgG2 (pMSA: *p* < 0.001, pPD: *p* < 0.001), the anti-αSyn IgG3 (pMSA: *p* < 0.001, pPD: *p* < 0.001) and the anti-αSyn IgG4 (pMSA: *p* < 0.001, pPD: *p* < 0.001) immune compartment (Figure 2C–E). The reductions in relative levels of anti-αSyn IgG2, IgG3, and IgG4 were found regardless of prodromal age stage (0–4 years and <4 years) (Table S3 and Figure S1). We did observe a significant difference in anti-αSyn IgM nAbs, however, the multiple comparison test did not identify differences between groups (Figure 2F). 

### 2.3. Total αSyn Levels in pMSA and pPD

**Figure 3:**
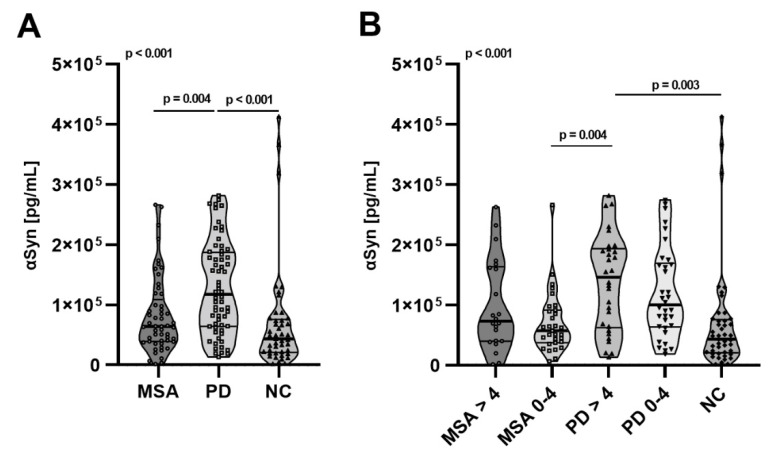
Serum total αSyn levels. (**A**) Distribution of total serum αSyn (pg/dL) in pMSA (*n* = 59), pPD (*n* = 82), and controls (*n* = 67), divided into prodromal age stages (>4 years prior to diagnosis) and (0–4 years prior to diagnosis) (**B**). Differences between groups were tested using multiple linear regression modeling including age and sex as confounding variables. Graphs are represented as truncated violin plots with median (bold lines) and 25% quantiles (thin lines).

Total amounts of serum αSyn were measured by MSD commercial assay. Interestingly, we observed an increased amount of total αSyn in pPD compared to pMSA (*p* = 0.004) and controls (*p* < 0.001) (Figure 3A). The significant increase was secluded to >4 years prior to diagnosis compared to both pMSA (*p* = 0.004) and controls (*p* < 0.001).

## 3. Discussion

This is the first study to report aberrancies in the anti-αSyn nAb compartment in prodromal stages of MSA and PD. We previously reported similar aberrancies in anti-αSyn nAbs in these two synucleinopathies as part of the disease progression in diagnosed patients [21,24]. Firstly, here we report both low-affinity and high-affinity repertoires of anti-αSyn nAbs in the serum of controls as well as prodromal MSA and PD cases, and as seen for diagnosed patients [21,24], the high-affinity anti-αSyn nAb repertoire was significantly reduced in pMSA and pPD (Figure 1). In pMSA, the reduction was found predominantly close to disease onset (0–4 years), whereas in pPD this decline was observed at earlier prodromal stages (>4 years). Secondly, we observed that pPD had increased relative levels of anti-αSyn IgG-total nAbs, very likely due to the increase in anti-αSyn IgG1 fraction (Figure 2), and there was a relative reduction of anti-αSyn IgG2, IgG3, and IgG4 levels in both pMSA and pPD compared to healthy controls. This is surprising, as it is opposite to that observed in already diagnosed MSA and PD patients [20,21]. 

Anti-αSyn nAbs have extensively been evaluated in blood plasma and to some extent also in cerebrospinal fluid in PD, MSA, and PD with dementia (PDD). A thorough and systemic meta-analysis by Scott et al. 2018 [22] found evidence for an increase in αSyn nAbs in PD patients particularly at early stages (<5.9 years) [19,26,27,28,29], which is in line with our results showing increases in anti-αSyn total IgG levels nAbs already in the prodromal phase of PD. Notably, the IgG reactivity is driven by the IgG1 subclass responses. IgG1 is the most abundant IgG subclass as it is considered to be a classical mediator, binding to every human Fc gamma receptor (FcγRs) on immune cells, leading to pro-inflammatory cytokine production, antibody-mediated phagocytosis, and C1q complement activation [30,31]. We speculate that the increase in anti-αSyn nAb responses is triggered by a higher load of peripheral αSyn in these patients, however, as the high-affinity antibody pool is reduced, this increased IgG1 production may be characterized by a lower affinity profile, resulting in diminished capacity for immune clearance leading to αSyn aggregation. In support of this, it was recently shown that anti-αSyn nAbs present in healthy individuals not only recognize different αSyn structures [32], but also inhibit the aggregation of αSyn by altering fibril formation in cell culture [33]. Further, anti-αSyn nAbs isolated from IVIg has shown rescuing effects in both low- and high-dose paradigms in the A53T transgenic PD mice model [17]. 

When looking at the IgGs subclasses, we observed decreases in anti-αSyn IgG2, IgG3, and IgG4 nAbs levels in PD and MSA compared to control individuals. These subclass-specific aberrancies prior to disease may suggest a compartmentalization discrepancy in the antibody-producing B cells. Supporting this, several studies have investigated B cells [34,35,36], most recently, in a single-cell study in PD patients, where they discovered a significant decrease in the naive B cell population, an increase in the memory B cells, and a higher frequency in immunoglobulin class switching in PD patients [37]. They furthermore showed upregulated MHC class II gene and AP-1 transcription factor expression, indicating an increased antigen-presentation in PD patients [37]. Furthermore, αSyn-specific T-cell reactivity in PD patients prior to diagnosis and around motor symptom initiation that decreases during disease progression, supports the idea that there are αSyn-specific antigen responses in early PD [38]. There is evidence that both the humoral and cell-mediated (adaptive) immunity compartments are implicated in prodromal and early PD stages [39] and likely also for MSA [40,41]. To what extent B-cells populations are also dysregulated in MSA must be investigated. An additional interesting tactic would be using the results from this study and others, not only for biomarker potential, but also in the debate regarding passive immunization candidates for synucleinopathies. Most candidates are of the IgG1 subclass [42]. The present results and previous reports show that especially MSA have increased IgG1 anti-αSyn nAbs in plasma and IgG1 and IgG3 in CSF [20,21]. The increments of these specific possibly pro-inflammatory nAbs could contribute to the ongoing inflammation, proposed to be initiated by the interplay between the immune system and aSyn [43]. The rationale behind choosing IgG4 for the ABBV-0805 candidate relies on the hypothesis to reduce inflammation by stabilizing the IgG4 molecule, which lacks the complement-binding functions and has a weaker Fcγ receptor interaction, resulting in less pro-inflammatory responses, which is beneficial for long-term immunization [44].

The pathological hallmarks of MSA and PD are the abnormal accumulation of the protein αSyn [1,2], but, on the other hand, αSyn is also a crucial protein involved in vesicle transport [45] and a key component of the immune system, playing a critical role in the development of both B cells and T cells, regulating immune function, as well as influencing microglia [46,47,48,49]. Hence, deregulation of αSyn levels may not only have a pathological effect reflected in the inclusions in neurons and oligodendrocytes, but also directly affect immune system regulation, which may influence the production of anti-αSyn nAbs. 

**Figure 4:**
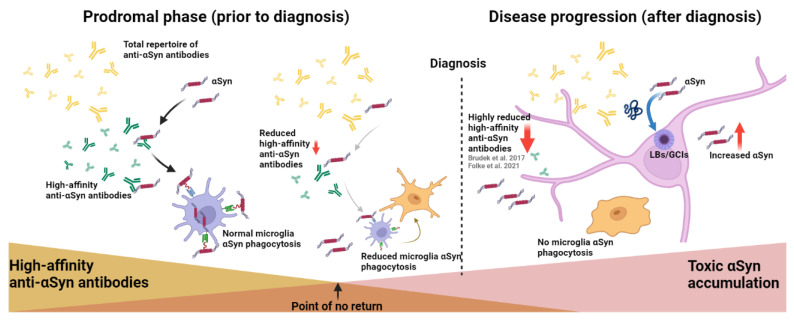
Proposed hypothesis of specific anti-αSyn nAbs decline by proxy facilitating an increase of αSyn following prodromal phases of MSA and PD and post diagnosis. nAbs, normally implicated in the clearing processes of excess amounts of αSyn inhibiting pathogenic species (oligomeric, protofibrils and fibril structures e.g., Lewy bodies (LBs) and Glial cytoplasmic inclusions (GCIs)) accumulation and interrupting αSyn seeding properties. Intervention with anti-αSyn nAbs could facilitate clearance of pathogenic αSyn.

Based on our results, we hypothesize that the immune clearance mechanism in MSA and PD is under pressure, probably already many years before disease onset, rendering subclinical pathological immune changes before symptomatic manifestations (Figure 4). We previously found the same observation in CSF samples of MSA and PD patients [21], which is suggestive that the autoimmune decline is not restricted only to the lymphatic system but also apparent in the CSF and possibly also in the brain. Hence, we believe that the autoimmune aberrancies are a whole-body disease phenomenon. That being said, whether the decline in CSF is present in the prodromal phases and whether the decline is apparent in the brain following disease progression and in the prodromal phases needs to be further elucidated. The important question that remains is if the reduction of high-affinity anti-αSyn-nAbs is causative to the toxic accumulation of αSyn or if the increasing load of αSyn applies a pressure to the immune system, tipping the balance towards an exhaustion of the immune response? 

Our study also brings up an important aspect of the length of the prodromal phases in both MSA and PD. Given the linear association (Figure 1K), it seems that the course of anti-αSyn specific autoimmune decline in the prodromal phase of MSA is shorter, about 4 years (Figure 1J), yet more progressive in comparison to pPD, which seems to have a slower autoimmune decline, extended over a longer prodromal period (Figure 1J). There are reports corroborating our findings and suggesting that the median time to phenoconversion from iRBD is 8.0 years for PD, with an overall phenoconversion rate of 6.25% per year and a suggested shorter period for MSA, with 16 patients eventually developing MSA after a mean follow-up time of 4.5 years [50]. Age at diagnosis of iRBD was 60 ± 8 years, and age at conversion to MSA 64 ± 9 years, with a time from iRBD diagnosis to conversion of 4 ± 3 years. Prodromal MSA and PD evidence, however, is still limited.

There are some limitations to discuss in this study. Although the sample size fulfills the recommended criteria for these types of studies [22], we must consider the biological variations, which manifested in relatively large distribution variations in our samples. Another limitation is the time-gap there may be between the clinical onset vs. definite diagnosis of particularly MSA patients, but also in some PD individuals, as these diseases may be masqueraded by other diseases at early stages [51]. So, we cannot rule out that some patient samples were taken when the patient already had shown the first clinical signs even though the final diagnosis was not given. 

The strength of this study relies in the unique cohort from the Danish National Biobank, which provides us with access to samples obtained from all individuals in contact to the primary health care service, which are stored in a biobank for future research. This is linked to the Danish National Patient Register, where all diagnoses are registered. These results are explorative, and the next step will be to validate levels of anti-αSyn antibodies in a longitudinal study. 

## 4. Materials and Methods

### 4.1. Patient Samples

A total of 208 serum samples were retrieved from the Danish National Biobank, Statens Serum Institute (SSI), Copenhagen, DK, which contains more than 25 million samples collected by the Danish public health sector and connected to the Danish National Patient Register. pMSA diagnosis was identified as: ICD-9 code 333 up to 1998 and with ICD-10 codes: G23 and G90.3 from 1999 to present. pPD was identified as: ICD-9 code 332 code up to 1998 and ICD-10 code G20 code from 1999 to present. The control samples were randomly chosen based on general background population cohort on negative diagnosis criteria in the following: Infectious and parasitic diseases: ICD-10: A00-B99; ICD-8: 000-136; diseases of the blood and blood-forming organs and certain disorders involving the immune mechanism: ICD10: D50-D89; ICD8: 280-289; diabetes: IDC10: E10-E11; ICD8: 249–250; diseases of the nervous system: ICD10: G00-G99; ICD8 320-358; diseases of the respiratory system: ICD10 J00-J99; ICD8 460-519; diseases of the skin and subcutaneous tissue: ICD10 L00-L99; ICD8 680-709; and diseases of the musculoskeletal system and connective tissue: ICD10 M00-M99; ICD8 710-738. We analyzed 59 pMSA, 82 pPD, and 67 healthy controls (Table 1). The project was approved by the Regional ethical committee (j.no.: H-15016232) and the Danish data protection agency (j.no.: P-2020-937). All demographic data were blinded to the experimental worker and first disclosed at analysis point. 

### 4.2. αSyn Affinity Measurement Using Electrochemiluminescence Assay

The evaluation of binding affinity was carried out as previously reported [24], adjusted to the ultrasensitive Meso Scale Discovery (MSD) platform. For the experiment, 96-well standard MSD plates (Meso Scale Diagnostics, LLC, Rockville, MD, USA, SD, cat# L15XA-1) were coated with 15 ng/mL αSyn (rPeptide, Watkinsville, GA, USA, cat# S1001-2) in ice-cold 0.1 M carbonate buffer (Sigma-Aldrich, St. Louis, MO, USA, cat# C3041) at 4 °C overnight (min. 12 h). The next day the plates were blocked with PBS + Bovine Serum Albumin (BSA) fraction V 3% + 0.1% TERGITOL surfactant (Merck, Darmstadt, Germany, cat# NP40S) in 150 µL/well for 2 h at 800 rpm at room temperature (RT). The plates were washed three times in 150 µL/well washing buffer (PBS + 0.05%Tween-20). For competition reaction, 30 µL of serum (1:100 in PBS + 0.1%BSA) were added to 1.4 mL U-bottom Micronic tubes (Micronic, Lelystad, The Netherlands, cat# MP226RN) with the subsequent addition of 30 µL of monomeric αSyn (rPeptide, Watkinsville, GA, USA, cat# S1001-2) in a range of concentrations: 2000 nM, 200 nM, 50 nM, 12.5 nM, 2 nM, and 0 nM and incubated for 1 h at RT. Following, 50 µL/well of the competition reactions were transferred onto plates and incubated for 1 h at RT. After three washes with 150 µL/well washing buffer, 25 µL/well of goat anti-human IgG SULFO-TAG antibody (Meso Scale Diagnostics, LLC, Rockville, MD, USA, cat# R32AJ-1; RRID:AB_2905663) (1:500 in PBS + BSA0.1%) was added and incubated on 800 rpm for 1 h at RT. Plates were washed for the last time and 150 µL/well MSD read buffer (Meso Scale Diagnostics, LLC, Rockville, MD, USA,, cat# R92TC) diluted 1:2 in milliQ water was added. Immediately after, the plates were read on MSD sector Imager S600 (Meso Scale Diagnostics, LLC, Rockville, MD, USA). 

### 4.3. nAbs-αSyn Subclass Measurements Using ELISA

The evaluation of IgG-total, IgG subclasses (IgG1–4), and IgM anti-αSyn nAbs was carried out as previously reported with minor adaptions to serum samples [20,21]. In brief, Nunc MaxiSorp 96-well plates (Thermo Scientific, Waltham, MA, USA, cat#439454) were coated with 5 µg/mL αSyn (rPeptide, Watkinsville, GA, USA, cat#S1001-2) in ice-cold 0.1 M carbonate buffer (Sigma-Aldrich, St. Louis, MO, USA, cat#C3041) at 4 °C overnight (min. 12 h). The next day, the plates were blocked with PBS + 3%BSA + 0.1%TERGITOL(Merck, Darmstadt, Germany, cat# NP40S) in 200 µL/well for 2 h at RT. Plates were washed three times with 150 µL/well washing buffer. Fifty microliters per well of diluted serum (1:100 in PBS + 0.1%BSA) were added and incubated for 1 h at RT. Afterwards, the plates were washed 3 times with 150 µL/well washing buffer. Fifty microliters per well of diluted secondary HRP-conjugated (IgG-total (1:30,000): Abcam, Cambridge, UK, cat#ab98595, RRID:AB_10673583) or biotin-conjugated antibodies (IgG1-4 and IgM) (IgG1 (1:1000): Invitrogen, Waltham, MA, USA, cat#MH1515, RRID:AB_2539710; IgG2 (1:5000): Sigma-Aldrich, St. Louis, MO, USA, cat#B3398, RRID:AB_258546; IgG3 (1:500): Sigma-Aldrich, St. Louis, MO, USA, cat#B3523, RRID:AB_258549; IgG4 (1:200): Sigma-Aldrich, St. Louis, MO, USA, cat#B3648, RRID:AB_258555; IgM (1:5000): Sigma-Aldrich, St. Louis, MO, USA, cat# B1265, RRID:AB_258514) were added and incubated for 1 h at RT. For the biotin-conjugated secondary antibodies, an additional step was carried out with streptavidin–peroxidase (Sigma-Aldrich, St. Louis, MO, USA, cat#S5512) for 30 min at RT. Finally, 50 µL/well of tetramethylbenzidine (TMB) substrate (Sigma-Aldrich, St. Louis, MO, USA, cat#T8665) was added and incubated in the dark for 30 min at RT. The enzymatic reaction was terminated by the addition of 0.5 N Sulfuric Acid (Merck, Darmstadt, Germany, cat#1.09073.1000) and the optical density was measured on a Multiscan^TM^ FC Microplate reader (Fischer Scientific^TM^, Waltham, MA, USA) at 450/620 nm. Negative controls included all conditions with the replacement of plasma with PBS + 0.1%BSA. All samples were normalized to a positive calibration control of a 2-fold serial dilution curve using the primary antibody against αSyn (1:10,000; Abcam, Cambridge, UK, cat#ab27766, RRID:AB_727020) and secondary goat anti-mouse-biotinylated antibody (1:1000; Vector Laboratories, Burlingame, CA, USA, cat#BA-9200). 

### 4.4. αSyn Measurement Using MSD Platform

Total αSyn in serum samples was measured using the commercially available U-PLEX^®^ Human αSyn kit (MSD^®^ Multi-Array Assay System, Meso Scale Diagnostics, LLC, Rockville, MD, USA, cat#K151WKK) following the manufacturer’s instructions. Prior to measurements, we optimized the assay to determine serum concentrations. Serum samples were diluted to 1:50 in Diluent 49 provided by MSD. The plates were measured at the MSD Sector Imager S600 (Meso Scale Diagnostics, LLC, Rockville, MD, USA). 

### 4.5. Statistics

The data were analyzed using R v. 3.5.2 [52] and GraphPad Prism v. 9.3.1 (GraphPad Software Inc., San Diego, CA, USA). Differences in demographics were tested using Fischer’s exact test for sex, Kruskall–Wallis test with Dunn’s multiple comparison test for age at sample withdrawal, and Mann–Whitney *U* test for Age at diagnosis and prodromal age (years prior to diagnosis). Outliers were excluded using the ROUT test with false discovery rate, Q, set at 1%. The percentage of maximum binding was calculated as following: % of max binding = 100 − (OD_read out_ − OD_2000nM competition_)/(OD_0nM competition_ − OD_2000nM competition_) × 100. For group comparison, we used multiple linear regression including age and sex as possible confounding variables using Anova from the car package [53]. For multiple comparisons using Tukey’s range test including confounding variables, the *glht* and *mcp* functions from the *multcomp* package [54] were applied. To determine associations with prodromal age and measured outcomes, Pearson’s correlation was used. Differences were considered significant at *p* < 0.05.

## 5. Conclusions

In conclusion, this study revealed that the decline in repertoire of high-affinity anti-αSyn nAbs in MSA and PD patients appears to be present already in the prodromal phases of MSA and PD. Furthermore, our study supports the supposition that the anti-αSyn IgG nAb responses in PD are dominant at early disease stages. This can be a classical IgG1 response to the increased serum concentration of circulating αSyn protein. These results further frame how immunopathogenesis may be a contributing factor to the etiology of synucleinopathies and thereby advocates for effective, passive immunizations already in prodromal or/and early stages of synucleinopathies. 

## Figures and Tables

**Table 1:** Demographic data of prodromal MSA, prodromal PD, and normal controls.

	pMSA (*n* = 59)	pPD (*n* = 82)	NC (*n* = 67)	*p*-Value
Sex, Female (%) ^§^	66.1	44.8	46.3	0.063
Age at sample, years ^#^	57.9 (16.9) (20–90) *	69.8 (11.5)(20–93)	61.7 (11.8) (28–89) *	<0.001
Age at diagnosis, years ^¤^	61.7 (16.5) (25–91)	74.3 (10.6) (23–93)	-	<0.001
Prodromal age, years ^¤^	3.8 (2.6) (0.6–10.1)	4.6 (3.1) (0.8–11.8)	-	0.113

^§^: Fisher’s exact test. ^#^: Kruskal–Wallis test with Dunn’s multiple comparison test. (): brackets describe the ranges from lowest to highest. *: *p* < 0.01 compared to PD. ^¤^: Mann–Whitney *U* test.

## Data Availability

Data can be accessed upon reasonable request.

## Supplementary Materials

The following supporting information can be downloaded at: https://www.mdpi.com/article/10.3390/ijms23126554/s1.

## References

[1] Spillantini M.G., Schmidt M.L., Lee V.M., Trojanowski J.Q., Jakes R., Goedert M. (1997). Alpha-synuclein in Lewy bodies. Nature.

[2] Spillantini M.G., Crowther R.A., Jakes R., Cairns N.J., Lantos P.L., Goedert M. (1998). Filamentous alpha-synuclein inclusions link multiple system atrophy with Parkinson’s disease and dementia with Lewy bodies. Neurosci. Lett..

[3] Goedert M., Jakes R., Spillantini M.G. (2017). The Synucleinopathies: Twenty Years on. J. Parkinson’s Dis..

[4] Doppler K., Ebert S., Uçeyler N., Trenkwalder C., Ebentheuer J., Volkmann J., Sommer C. (2014). Cutaneous neuropathy in Parkinson’s disease: A window into brain pathology. Acta Neuropathol..

[5] Doppler K., Weis J., Karl K., Ebert S., Ebentheuer J., Trenkwalder C., Klebe S., Volkmann J., Sommer C. (2015). Distinctive distribution of phospho-alpha-synuclein in dermal nerves in multiple system atrophy. Mov. Disord..

[6] Beach T.G., Serrano G.E., Kremer T., Canamero M., Dziadek S., Sade H., Derkinderen P., Corbillé A.-G., Letournel F., Munoz D.G. (2018). Immunohistochemical Method and Histopathology Judging for the Systemic Synuclein Sampling Study (S4). J. Neuropathol. Exp. Neurol..

[7] Wakabayashi K., Takahashi H., Ohama E., Ikuta F. (1990). Parkinson’s disease: An immunohistochemical study of Lewy body-containing neurons in the enteric nervous system. Acta Neuropathol..

[8] Qualman S.J., Haupt H.M., Yang P., Hamilton S.R. (1984). Esophageal Lewy bodies associated with ganglion cell loss in achalasia. Similarity to Parkinson’s disease. Gastroenterology.

[9] Kuzkina A., Schulmeyer L., Monoranu C.-M., Volkmann J., Sommer C., Doppler K. (2019). The aggregation state of α-synuclein deposits in dermal nerve fibers of patients with Parkinson’s disease resembles that in the brain. Parkinsonism Relat. Disord..

[10] Wang Z., Becker K., Donadio V., Siedlak S., Yuan J., Rezaee M., Incensi A., Kuzkina A., Orrú C.D., Tatsuoka C. (2021). Skin α-Synuclein Aggregation Seeding Activity as a Novel Biomarker for Parkinson Disease. JAMA Neurol..

[11] Challis C., Hori A., Sampson T.R., Yoo B.B., Challis R.C., Hamilton A.M., Mazmanian S.K., Volpicelli-Daley L.A., Gradinaru V. (2020). Gut-seeded α-synuclein fibrils promote gut dysfunction and brain pathology specifically in aged mice. Nat. Neurosci..

[12] Maass F., Rikker S., Dambeck V., Warth C., Tatenhorst L., Csoti I., Schmitz M., Zerr I., Leha A., Bähr M. (2020). Increased alpha-synuclein tear fluid levels in patients with Parkinson’s disease. Sci. Rep..

[13] Hamm-Alvarez S.F., Janga S.R., Edman M.C., Feigenbaum D., Freire D., Mack W.J., Okamoto C.T., Lew M.F. (2019). Levels of oligomeric α-Synuclein in reflex tears distinguish Parkinson’s disease patients from healthy controls. Biomark. Med..

[14] Abd Elhadi S., Grigoletto J., Poli M., Arosio P., Arkadir D., Sharon R. (2019). α-Synuclein in blood cells differentiates Parkinson’s disease from healthy controls. Ann. Clin. Transl. Neurol..

[15] Lutz H.U. (2007). Homeostatic roles of naturally occurring antibodies: An overview. J. Autoimmun..

[16] Lutz H.U. (2012). Naturally Occurring Antibodies (NAbs).

[17] Huang Y.-R., Xie X.-X., Ji M., Yu X.-L., Zhu J., Zhang L.-X., Liu X.-G., Wei C., Li G., Liu R.-T. (2019). Naturally occurring autoantibodies against α-synuclein rescues memory and motor deficits and attenuates α-synuclein pathology in mouse model of Parkinson’s disease. Neurobiol. Dis..

[18] Weihofen A., Liu Y., Arndt J.W., Huy C., Quan C., Smith B.A., Baeriswyl J.-L., Cavegn N., Senn L., Su L. (2019). Development of an aggregate-selective, human-derived α-synuclein antibody BIIB054 that ameliorates disease phenotypes in Parkinson’s disease models. Neurobiol. Dis..

[19] Smith L.M., Schiess M.C., Coffey M.P., Klaver A.C., Loeffler D.A. (2012). α-Synuclein and Anti-α-Synuclein Antibodies in Parkinson’s Disease, Atypical Parkinson Syndromes, REM Sleep Behavior Disorder, and Healthy Controls. PLoS ONE.

[20] Folke J., Rydbirk R., Løkkegaard A., Salvesen L., Hejl A.-M., Starhof C., Bech S., Winge K., Christensen S., Pedersen L.Ø. (2019). Distinct Autoimmune Anti-α-Synuclein Antibody Patterns in Multiple System Atrophy and Parkinson’s Disease. Front. Immunol..

[21] Folke J., Rydbirk R., Løkkegaard A., Hejl A.-M., Winge K., Starhof C., Salvesen L., Pedersen L.Ø., Aznar S., Pakkenberg B. (2021). Cerebrospinal fluid and plasma distribution of anti-α-synuclein IgMs and IgGs in multiple system atrophy and Parkinson’s disease. Parkinsonism Relat. Disord..

[22] Scott K.M., Kouli A., Yeoh S.L., Clatworthy M.R., Williams-Gray C.H. (2018). A Systematic Review and Meta-Analysis of Alpha Synuclein Auto-Antibodies in Parkinson’s Disease. Front. Neurol..

[23] Vidarsson G., Dekkers G., Rispens T. (2014). IgG subclasses and allotypes: From structure to effector functions. Front. Immunol..

[24] Brudek T., Winge K., Folke J., Christensen S., Fog K., Pakkenberg B., Pedersen L.O. (2017). Autoimmune antibody decline in Parkinson’s disease and Multiple System Atrophy; a step towards immunotherapeutic strategies. Mol. Neurodegener..

[25] de Natale E.R., Wilson H., Politis M. (2022). Predictors of RBD progression and conversion to synucleinopathies. Curr. Neurol. Neurosci. Rep..

[26] Horvath I., Iashchishyn I.A., Forsgren L., Morozova-Roche L.A. (2017). Immunochemical Detection of α-Synuclein Autoantibodies in Parkinson’s Disease: Correlation between Plasma and Cerebrospinal Fluid Levels. ACS Chem. Neurosci..

[27] Yanamandra K., Gruden M.A., Casaite V., Meskys R., Forsgren L., Morozova-Roche L.A. (2011). Alpha-Synuclein Reactive Antibodies As Diagnostic Biomarkers in Blood Sera of Parkinson’s Disease Patients. PLoS ONE.

[28] Shalash A., Salama M., Makar M., Roushdy T., Elrassas H.H., Mohamed W., El-Balkimy M., Donia M.A. (2017). Elevated serum α-synuclein autoantibodies in patients with parkinson’s disease relative to Alzheimer’s disease and controls. Front. Neurol..

[29] Xu Q., Evetts S., Hu M., Talbot K., Wade-Martins R., Davis J.J. (2014). An impedimetric assay of α-synuclein autoantibodies in early stage Parkinson’s disease. RSC Adv..

[30] Bruhns P., Iannascoli B., England P., Mancardi D.A., Fernandez N., Jorieux S., Daëron M. (2009). Specificity and affinity of human Fcγ receptors and their polymorphic variants for human IgG subclasses. Blood.

[31] Hayes J.M., Wormald M.R., Rudd P.M., Davey G.P. (2016). Fc gamma receptors: Glycobiology and therapeutic prospects. J. Inflamm. Res..

[32] Albus A., Gold M., Bach J.-P., Burg-Roderfeld M., Jördens M., Kirchhein Y., Kronimus Y., Mengel D., Zerr I., Dodel R. (2018). Extending the functional characteristics of naturally occurring autoantibodies against β-Amyloid, Prion Protein and α-Synuclein. PLoS ONE.

[33] Braczynski A.K., Sevenich M., Gering I., Kupreichyk T., Agerschou E.D., Kronimus Y., Habib P., Stoldt M., Willbold D., Schulz J.B. (2022). Alpha-Synuclein-Specific Naturally Occurring Antibodies Inhibit Aggregation In Vitro and In Vivo. Biomolecules.

[34] Li R., Tropea T.F., Baratta L.R., Zuroff L., Diaz-Ortiz M.E., Zhang B., Shinoda K., Rezk A., Alcalay R.N., Chen-Plotkin A. (2022). Abnormal B-Cell and Tfh-Cell Profiles in Patients with Parkinson Disease: A Cross-sectional Study. Neurol.-Neuroimmunol. NeuroInflamm..

[35] Bas J., Calopa M., Mestre M., Molleví D.G., Cutillas B., Ambrosio S., Buendia E. (2001). Lymphocyte populations in Parkinson’s disease and in rat models of parkinsonism. J. Neuroimmunol..

[36] Stevens C.H., Rowe D., Morel-Kopp M.-C., Orr C., Russell T., Ranola M., Ward C., Halliday G.M. (2012). Reduced T helper and B lymphocytes in Parkinson’s disease. J. Neuroimmunol..

[37] Wang P., Luo M., Zhou W., Jin X., Xu Z., Yan S., Li Y., Xu C., Cheng R., Huang Y. (2022). Global Characterization of Peripheral B Cells in Parkinson’s Disease by Single-Cell RNA and BCR Sequencing. Front. Immunol..

[38] Lindestam Arlehamn C.S., Dhanwani R., Pham J., Kuan R., Frazier A., Rezende Dutra J., Phillips E., Mallal S., Roederer M., Marder K.S. (2020). α-Synuclein-specific T cell reactivity is associated with preclinical and early Parkinson’s disease. Nat. Commun..

[39] Contaldi E., Magistrelli L., Comi C.T. (2022). Lymphocytes in Parkinson’s Disease. J. Parkinson’s Dis..

[40] Williams G.P., Marmion D.J., Schonhoff A.M., Jurkuvenaite A., Won W.-J., Standaert D.G., Kordower J.H., Harms A.S.T. (2020). cell infiltration in both human multiple system atrophy and a novel mouse model of the disease. Acta Neuropathol..

[41] Cao B., Chen X., Zhang L., Wei Q., Liu H., Feng W., Chen Y., Shang H. (2020). Elevated Percentage of CD3+ T-Cells and CD4+/CD8+ Ratios in Multiple System Atrophy Patients. Front. Neurol..

[42] Folke J., Ferreira N., Brudek T., Borghammer P., Van Den Berge N. (2022). Passive Immunization in Alpha-Synuclein Preclinical Animal Models. Biomolecules.

[43] Allen Reish H.E., Standaert D.G. (2015). Role of α-synuclein in inducing innate and adaptive immunity in Parkinson disease. J. Parkinson’s Dis..

[44] Nordström E., Eriksson F., Sigvardson J., Johannesson M., Kasrayan A., Jones-Kostalla M., Appelkvist P., Söderberg L., Nygren P., Blom M. (2021). ABBV-0805, a novel antibody selective for soluble aggregated α-synuclein, prolongs lifespan and prevents buildup of α-synuclein pathology in mouse models of Parkinson’s disease. Neurobiol. Dis..

[45] Huang M., Wang B., Li X., Fu C., Wang C., Kang X. (2019). α-Synuclein: A Multifunctional Player in Exocytosis, Endocytosis, and Vesicle Recycling. Front. Neurosci..

[46] Shameli A., Xiao W., Zheng Y., Shyu S., Sumodi J., Meyerson H.J., Harding C.V., Maitta R.W. (2016). A critical role for alpha-synuclein in development and function of T lymphocytes. Immunobiology.

[47] Xiao W., Shameli A., Harding C.V., Meyerson H.J., Maitta R.W. (2014). Late stages of hematopoiesis and B cell lymphopoiesis are regulated by α-synuclein, a key player in Parkinson’s disease. Immunobiology.

[48] Kim C., Lee H.-J., Masliah E., Lee S.-J. (2016). Non-cell-autonomous Neurotoxicity of α-synuclein Through Microglial Toll-like Receptor 2. Exp. Neurobiol..

[49] Alam M.M., Yang D., Li X.-Q., Liu J., Back T.C., Trivett A., Karim B., Barbut D., Zasloff M., Oppenheim J.J. (2022). Alpha synuclein, the culprit in Parkinson disease, is required for normal immune function. Cell Rep..

[50] Postuma R.B., Iranzo A., Hu M., Högl B., Boeve B.F., Manni R., Oertel W.H., Arnulf I., Ferini-Strambi L., Puligheddu M. (2019). Risk and predictors of dementia and parkinsonism in idiopathic REM sleep behaviour disorder: A multicentre study. Brain.

[51] Koga S., Aoki N., Uitti R.J., Van Gerpen J.A., Cheshire W.P., Josephs K.A., Wszolek Z.K., Langston J.W., Dickson D.W. (2015). When DLB, PD, and PSP masquerade as MSA. Neurology.

[52] Team R.C. (2014). R: A Language and Environment for Statistical Computing.

[53] Fox J., Weisberg S. (2011). An R Companion to Applied Regression.

[54] Hothorn T., Bretz F., Westfall P. (2008). Simultaneous inference in general parametric models. Biom. J..

