# Supplementary material for: Alpha-Synuclein Autoimmune Decline in Prodromal Multiple System Atrophy and Parkinson’s Disease
Source: Int J Mol Sci. 2022 Jun 12;23(12):6554. doi: 10.3390/ijms23126554 (PMC9224313; doi:10.3390/ijms23126554)
Supplement: Supplementary file 1 [file ijms-23-06554-s001.zip › ijms-1762458-supplementary.pdf]

# Supplementary materials:

**Title:**
Alpha-synuclein Autoimmune Decline in Prodromal Multiple System Atrophy and Parkinson's Disease

**Authors and affiliations:**
Jonas Folke, PhD[1,2,*,#], Emil Bergholt, MSc[1,*], Bente Pakkenberg, MD, DMSc[1,3], Susana Aznar, PhD[1,2], and Tomasz Brudek, PhD[1,2].

*: JF and EB shares first authorship.

[1]: Centre for Neuroscience and Stereology, Department of Neurology, Bispebjerg-Frederiksberg Hospital, University Hospital of Copenhagen, DK-2400 Copenhagen NV.

[2]: Copenhagen Center for Translational Research, Bispebjerg-Frederiksberg Hospital, University Hospital of Copenhagen, DK-2400 Copenhagen NV.

[3]: Faculty of Health and Medical Sciences, University of Copenhagen, DK-2200 Copenhagen N.

**[#]: Corresponding author:**
Jonas Folke, PhD
Centre for Neuroscience and Stereology,
Department of Neurology,
Bispebjerg-Frederiksberg Hospital,
University Hospital of Copenhagen,
Nielsine Nielsens Vej 6B, Entrance 11B, 2. floor,
2400 Copenhagen NV, Denmark.
Phone: +45 38636113
E-mail: Jonas.folke@regionh.dk

**Supplementary material:** 3 tables and 1 figure.

## Supplementary Tables:

**Table S1: Complete statistics of prodromal(p) Multiple System Atrophy (pMSA), Parkinson's Disease (pPD) and normal controls (NC).**

| | Model statistics | | | Multiple comparison test | | | PD vs MSA | NC vs MSA | NC vs PD |
|---|---|---|---|---|---|---|---|---|---|
| | **F-statistics** | $R^2$ | **p-value** | **p-value (Group)** | **p-value (Age)** | **p-value (Sex)** | **p-value** | **p-value** | **p-value** |
| **αSyn Competition** | | | | | | | | | |
| nM200 | F(4,192)=0.49 | -0.01 | 0.746 | 0.463 | 0.879 | 0.912 | - | - | - |
| nM50 | F(4,184)=1.09 | 0.01 | 0.363 | 0.379 | 0.203 | 0.344 | - | - | - |
| nM12.5 | F(4,176)=3.73 | 0.06 | 0.006 | **0.002** | 0.650 | 0.256 | 0.779 | **0.004** | **0.020** |
| nM2 | F(4,161)=5.90 | 0.11 | 0.0002 | **9.0E-05** | 0.400 | 0.484 | 0.576 | **0.011** | **<0.001** |
| **Anti-αSyn nAbs subclasses** | | | | | | | | | |
| IgG-T | F(4,202)=4.64 | 0.07 | 0.001 | **3.0E-04** | 0.777 | 0.745 | **0.001** | 0.936 | **0.002** |
| IgG1 | F(4,195)=3.32 | 0.05 | 0.012 | **0.006** | 0.644 | 0.533 | **0.018** | 0.984 | **0.017** |
| IgG2 | F(4,185)=18.0 | 0.27 | 1.6E-12 | **2.7E-13** | 0.150 | 0.933 | 0.609 | **<0.001** | **<0.001** |
| IgG3 | F(4,200)=11.3 | 0.17 | 2.6E-08 | **2.8E-09** | 0.551 | 0.976 | **<0.001** | **<0.001** | 0.611 |
| IgG4 | F(4,203)=10.3 | 0.15 | 1.4E-07 | **1.2E-06** | 0.572 | **0.005** | 0.999 | **<0.001** | **<0.001** |
| IgM | F(4,198)=2.17 | 0.02 | 0.073 | **0.045** | **0.044** | 0.349 | 0.137 | 0.979 | **0.054** |
| **Total αSyn amount** | | | | | | | | | |
| αSyn | F(4,157)=5.63 | 0.10 | 0.0003 | **3.0E-04** | 0.476 | 0.092 | **0.004** | 0.868 | **<0.001** |

**Table S2: Correlations between prodromal age and measure outcome in prodromal(p) Multiple System Atrophy (pMSA), Parkinson's Disease (pPD) and normal controls (NC).**

| | MSA | | | | PD | | | |
|---|---|---|---|---|---|---|---|---|
| | ***r*** | ***R²*** | **95%CI** | **p-value** | ***r*** | ***R²*** | **95%CI** | **p-value** |
| **αSyn Competition** | | | | | | | | |
| nM200 | 0.096 | 0.009 | -0.17 to 0.35 | 0.472 | 0.012 | 0.001 | -0.21 to 0.23 | 0.917 |
| nM50 | -0.182 | 0.033 | -0.43 to 0.09 | 0.189 | -0.083 | 0.007 | -0.30 to 0.14 | 0.472 |
| nM12.5 | -0.025 | 0.001 | -0.30 to 0.25 | 0.858 | -0.209 | 0.044 | -0.42 to 0.03 | 0. 080 |
| nM2 | **-0.367** | **0.135** | **-0.08 to -0.37** | **0.014** | 0.014 | 0.001 | -0.23 to 0.26 | 0.914 |
| **Anti-αSyn nAbs subclasses** | | | | | | | | |
| IgG-T | -0.060 | 0.004 | -0.31 to 0.20 | 0.651 | -0.013 | 0.001 | -0.23 to 0.20 | 0.906 |
| IgG1 | 0.090 | 0.008 | -0.18 to 0.35 | 0.507 | -0.148 | 0.022 | -0.35 to 0.07 | 0.186 |
| IgG2 | -0.066 | 0.004 | -0.32 to 0.20 | 0.624 | 0.115 | 0.013 | -0.13 to 0.35 | 0.361 |
| IgG3 | 0.083 | 0.006 | -0.18 to 0.34 | 0.541 | 0.062 | 0.004 | -0.16 to 0.28 | 0.579 |
| IgG4 | -0.042 | 0.002 | -0.30 to 0.22 | 0.750 | 0.097 | 0.009 | -0.12 to 0.31 | 0.388 |
| IgM | 0.236 | 0.055 | -0.03 to 0.47 | 0.086 | -0.020 | 0.001 | -0.24 to 0.20 | 0.858 |
| **Total αSyn amount** | | | | | | | | |
| αSyn | 0.187 | 0.04 | -0.09 to 0.44 | 0.179 | 0.239 | 0.057 | -0.01 to 0.46 | 0.061 |

**Table S3: Complete statistics of prodromal individuals divided into longer period prior to diagnosis (> 4 years) and close to diagnosis (0-4 years) in pMSA, pPD and normal controls (NC).**

| | Model statistics | | | Multiple comparison test | | | MSA>4 vs MSA<4 | PD <4 vs MSA<4 | PD>4 vs MSA <4 | NC vs MSA<4 | PD<4 vs MSA>4 | PD>4 vs MSA>4 | NC vs MSA>4 | PD>4 vs PD<4 | NC vs PD<4 | NC vs PD>4 |
|---|---|---|---|---|---|---|---|---|---|---|---|---|---|---|---|---|
| | **F-statistics** | $R^2$ | **p-value** | **p-value (Group)** | **p-value (Sex)** | **p-value (Age)** | **p-value** | **p-value** | **p-value** | **p-value** | **p-value** | **p-value** | **p-value** | **p-value** | **p-value** | **p-value** |
| **αSyn Competition** | | | | | | | | | | | | | | | | |
| nM200 | F(6,190)=0.36 | -0.02 | 0.902 | 0.776 | 0.915 | 0.913 | - | - | - | - | - | - | - | - | - | - |
| nM50 | F(6,182)=0.79 | 0.00 | 0.576 | 0.668 | 0.222 | 0.358 | - | - | - | - | - | - | - | - | - | - |
| nM12.5 | F(6,174)=2.90 | 0.06 | 0.010 | **0.006** | 0.853 | 0.237 | 1.000 | 0.999 | 0.701 | **0.043** | 0.999 | 0.828 | 0.126 | 0.515 | **0.017** | 0.624 |
| nM2 | F(6,159)=4.05 | 0.10 | 0.0008 | **7.0E-04** | 0.463 | 0.501 | 0.887 | 0.992 | 0.997 | **0.027** | 0.699 | 0.742 | 0.559 | 0.999 | **0.0039** | **0.0042** |
| **Anti-αSyn nAbs subclasses** | | | | | | | | | | | | | | | | |
| IgG-T | F(6,200)=3.46 | 0.07 | 0.003 | **0.001** | 0.642 | 0.735 | 0.572 | 0.221 | 0.206 | 0.990 | **0.010** | **0.007** | 0.734 | 1.000 | **0.029** | **0.026** |
| IgG1 | F(6,193)=2.46 | 0.04 | 0.026 | **0.021** | 0.729 | 0.497 | 0.984 | **0.040** | 0.316 | 0.991 | 0.246 | 0.746 | 0.999 | 0.813 | **0.035** | 0.394 |
| IgG2 | F(6,183)=12.1 | 0.26 | 2E-11 | **6.2E-12** | 0.169 | 0.952 | 0.973 | 1.000 | 0.929 | **<0.001** | 0.956 | 0.682 | **<0.001** | 0.961 | **<0.001** | **<0.001** |
| IgG3 | F(6,198)=8.18 | 0.17 | 6.5E-08 | **1.2E-08** | 0.740 | 0.928 | 0.999 | **0.022** | **<0.001** | **<0.001** | **0.032** | **<0.001** | **<0.001** | 0.351 | 0.365 | 0.999 |
| IgG4 | F(6,201)=7.10 | 0.15 | 7.2E-07 | **8.8E-06** | 0.528 | **0.005** | 0.965 | 0.936 | 0.999 | **0.006** | 0.999 | 0.912 | **0.002** | 0.823 | **<0.001** | **0.007** |
| IgM | F(6,196)=1.48 | 0.01 | 0.188 | 0.170 | **0.040** | 0.370 | - | - | - | - | - | - | - | - | - | - |
| **Total αSyn amount** | | | | | | | | | | | | | | | | |
| aSyn | F(6,155)=4.28 | 0.11 | 0.0005 | **8.0E-04** | 0.306 | 0.105 | 0.596 | 0.084 | **0.004** | 0.999 | 0.902 | 0.382 | 0.675 | 0.854 | 0.074 | **0.003** |

**Supplementary figures:**

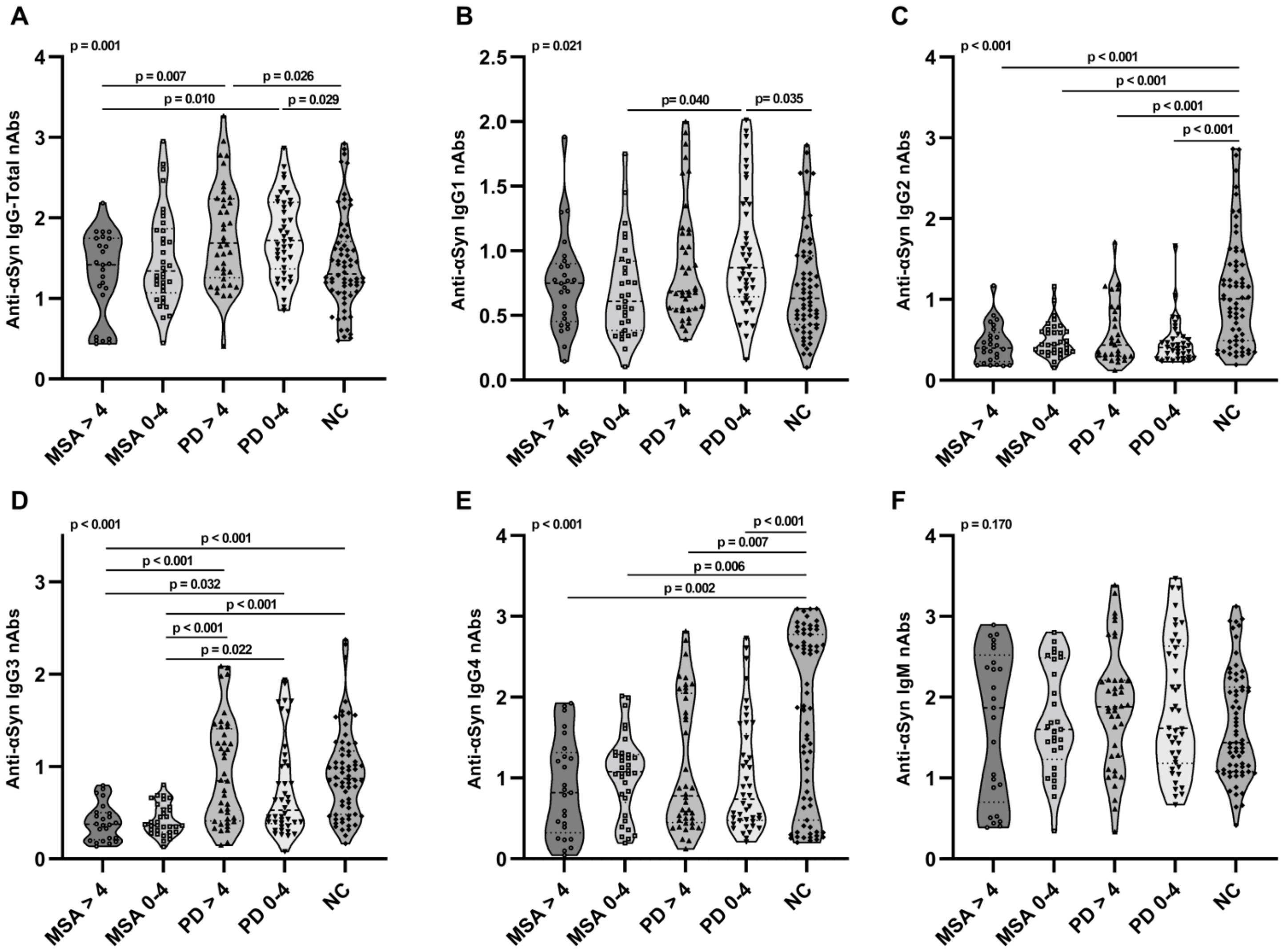


**Figure S1.** Serum nAbs affinity-profile towards αSyn. Binding of anti-αSyn IgG-total, IgG1-4 subclasses and IgM nAbs in individual pMSA (n = 59), pPD (n = 82) and controls (n = 67) for IgG serum fractions to αSyn in indirect ELISA assay. pMSA and pPD were divided into two prodromal age ranges (0-4 years prior to diagnosis and above 4 years prior to diagnosis) detected for (**A**) anti-αSyn IgG-total, (**B**) anti-αSyn IgG1, (**C**) anti-αSyn IgG2, (**D**) anti-αSyn IgG3, (**E**) anti-αSyn IgG4 and (**F**) anti-αSyn IgM. Differences between groups were tested using multiple linear regression modeling including age and sex as confounding variables. Graphs are represented as truncated violin plots with median and 25% quantiles.
